# 2-(Adamantan-1-yl)-5-(4-nitro­phen­yl)-1,3,4-oxadiazole

**DOI:** 10.1107/S1600536812005302

**Published:** 2012-02-24

**Authors:** Ali A. El-Emam, Adnan A. Kadi, Nasser R. El-Brollosy, Seik Weng Ng, Edward R. T. Tiekink

**Affiliations:** a Department of Pharmaceutical Chemistry, College of Pharmacy, King Saud University, Riyadh 11451, Saudi Arabia; b Department of Chemistry, University of Malaya, 50603 Kuala Lumpur, Malaysia; c Chemistry Department, Faculty of Science, King Abdulaziz University, PO Box 80203 Jeddah, Saudi Arabia

## Abstract

The title mol­ecule, C_18_H_19_N_3_O_3_, lies on a mirror plane that bis­ects the adamantyl group. In the crystal, C—H⋯O and C—H⋯N inter­actions lead to supra­molecular chains along [100]. These assemble into layers in the *ab* plane *via* π–π inter­actions [centroid–centroid distance = 3.6548 (7) Å] between the oxadiazole and benzene rings.

## Related literature

For the biological activity of adamantyl-1,3,4-oxadiazole derivatives, see: Kadi *et al.* (2007 ▶); El-Emam *et al.* (2004 ▶). For related adamantane structures, see: Al-Tamimi *et al.* (2010 ▶); Kadi *et al.* (2011 ▶).
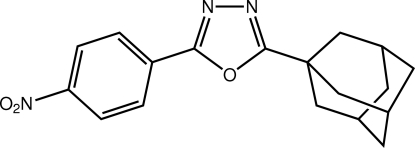


## Experimental

### 

#### Crystal data

C_18_H_19_N_3_O_3_
*M* *_r_* = 325.36
Monoclinic, 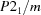

*a* = 6.8502 (6) Å
*b* = 6.5705 (7) Å
*c* = 17.6761 (15) Å
β = 98.432 (8)°
*V* = 786.99 (13) Å^3^
*Z* = 2
Mo *K*α radiation
μ = 0.10 mm^−1^
*T* = 100 K
0.30 × 0.30 × 0.15 mm

#### Data collection

Agilent SuperNova Dual diffractometer with an Atlas detector
Absorption correction: multi-scan (*CrysAlis PRO*; Agilent, 2011 ▶) *T*_min_ = 0.972, *T*_max_ = 0.986
3236 measured reflections
1956 independent reflections
1456 reflections with *I* > 2σ(*I*)
*R*_int_ = 0.027

#### Refinement

*R*[*F* ^2^ > 2σ(*F* ^2^)] = 0.047
*wR*(*F* ^2^) = 0.123
*S* = 1.05
1956 reflections
136 parameters
H-atom parameters constrained
Δρ_max_ = 0.30 e Å^−3^
Δρ_min_ = −0.34 e Å^−3^

### 

Data collection: *CrysAlis PRO* (Agilent, 2011 ▶); cell refinement: *CrysAlis PRO*; data reduction: *CrysAlis PRO*; program(s) used to solve structure: *SHELXS97* (Sheldrick, 2008 ▶); program(s) used to refine structure: *SHELXL97* (Sheldrick, 2008 ▶); molecular graphics: *ORTEP-3* (Farrugia, 1997 ▶) and *DIAMOND* (Brandenburg, 2006 ▶); software used to prepare material for publication: *publCIF* (Westrip, 2010 ▶).

## Supplementary Material

Crystal structure: contains datablock(s) global, I. DOI: 10.1107/S1600536812005302/gg2074sup1.cif

Structure factors: contains datablock(s) I. DOI: 10.1107/S1600536812005302/gg2074Isup2.hkl

Supplementary material file. DOI: 10.1107/S1600536812005302/gg2074Isup3.cml

Additional supplementary materials:  crystallographic information; 3D view; checkCIF report

## Figures and Tables

**Table 1:** Hydrogen-bond geometry (Å, °)

*D*—H⋯*A*	*D*—H	H⋯*A*	*D*⋯*A*	*D*—H⋯*A*
C13—H13*A*⋯N2^i^	0.95	2.59	3.297 (3)	132
C16—H16*A*⋯O2^ii^	0.95	2.49	3.256 (3)	137

Symmetry codes: (i) 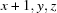; (ii) 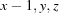.

**Fig. 1.**
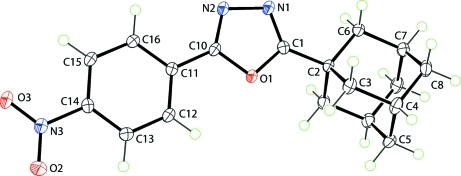
The molecular structure of (I) showing the atom-labelling scheme and displacement ellipsoids at the 50% probability level.

**Fig. 2.**
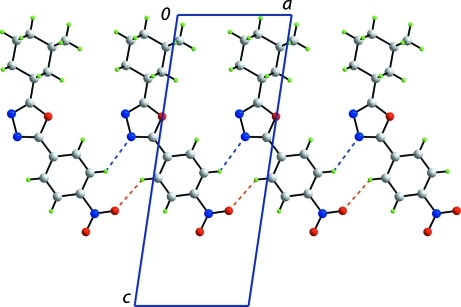
A view of the linear supramolecular chain along [100] in (I). The C—H···O and C—H···N interactions are shown as orange and blue dashed lines, respectively.

**Fig. 3.**
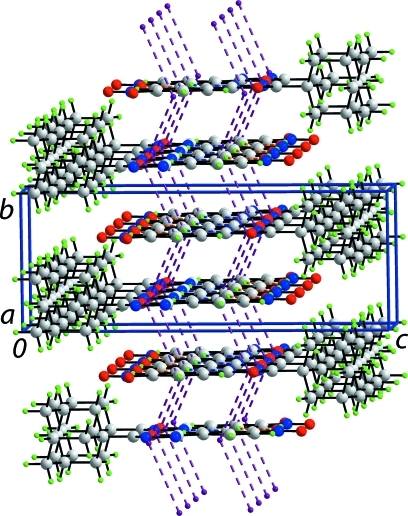
A view of a supramolecular layer in (I) whereby the chains illustrated in Fig. 2 are linked by π–π interactions (purple dashed lines).

**Fig. 4.**
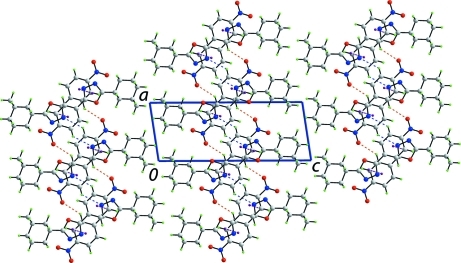
A view in projection along the b axis of the unit-cell contents for (I), highlighting the stacking of layers.

## supplementary crystallographic information

### Comment

Adamantane derivatives have been reported to exhibit marked anti-bacterial and anti-inflammatory activities (Kadi *et al.*, 2007; El-Emam *et al.*, 2004). In continuation of our interest in the chemical and pharmacological properties of adamantane derivatives, and as part of on-going structural studies of adamantane derivatives (Kadi *et al.*, 2011; Al-Tamimi *et al.*, 2010), the title compound, (I), was prepared as a potential chemotherapeutic agent.

The molecule of (I), Fig. 1, lies on a mirror plane that bisects the adamantyl residue. The molecules are linked into a supramolecular linear chains along [100] *via* C—H···O and C—H···N interactions, Fig. 2 and Table 1. The aforementioned interactions lead to 10-membered {···HC_2_NO···HC_3_N} synthons. The chains are linked into layers in the *ab* plane by π–π interactions occurring between the oxadiazole and phenyl rings [centroid···centroid distance = 3.6548 (7) Å, angle between rings = 0° for symmetry operation 2 - *x*, -1/2 + *y*, 1 - *z*]. Layers stack along the *b* axis with no specific interactions between them.

### Experimental

The title compound was prepared following our previously described method (Kadi *et al.*, 2007). A mixture of 4-nitrobenzoic acid hydrazide (1.81 g, 0.01 mol), 1-adamantane carboxylic acid (1.8 g, 0.01 mol) and phosphorus oxychloride (8 ml) was heated under reflux for 1 h. On cooling, crushed ice (50 g) was added and the mixture was stirred for 30 min. The separated crude product was filtered, washed with water, then with saturated sodium hydrogen carbonate solution and finally with water, dried and crystallized from EtOH/CHCl_3_ to yield 2.96 g (91%) of the title compound as colourless crystals. *M*.pt.: 511–513 K. ^1^H NMR (CDCl_3_): δ 1.79 (s, 6H, adamantane-H), 2.08 (s, 9H, adamantane-H), 7.61 (d, 2H, Ar—H, J = 8.3 Hz), 8.33 (d, 2H, Ar—H, J = 8.3 Hz). ^13^C NMR: δ 27.10, 33.15, 36.80, 39.82 (adamantane-C), 125.15, 128.20, 141.95, 145.10 (Ar—C), 163.25 (oxadiazole C-5), 173.05 (oxadiazole C-2).

### Refinement

Carbon-bound H-atoms were placed in calculated positions [C—H = 0.95 to 1.00 Å, *U*_iso_(H) = 1.2*U*_eq_(C)] and were included in the refinement in the riding model approximation. The (4 0 3) reflection was omitted owing to poor agreement.

### Crystal data

**Table:**

C_18_H_19_N_3_O_3_	*F*(000) = 344
*M**_r_* = 325.36	*D*_x_ = 1.373 Mg m^−^^3^
Monoclinic, *P*2_1_/*m*	Melting point: 512 K
Hall symbol: -P 2yb	Mo *K*α radiation, λ = 0.71073 Å
*a* = 6.8502 (6) Å	Cell parameters from 1102 reflections
*b* = 6.5705 (7) Å	θ = 3.0–27.5°
*c* = 17.6761 (15) Å	µ = 0.10 mm^−^^1^
β = 98.432 (8)°	*T* = 100 K
*V* = 786.99 (13) Å^3^	Prism, colourless
*Z* = 2	0.30 × 0.30 × 0.15 mm

### Data collection

**Table:**

Agilent SuperNova Dual diffractometer with an Atlas detector	1956 independent reflections
Radiation source: SuperNova (Mo) X-ray Source	1456 reflections with *I* > 2σ(*I*)
Mirror monochromator	*R*_int_ = 0.027
Detector resolution: 10.4041 pixels mm^-1^	θ_max_ = 27.6°, θ_min_ = 3.0°
ω scan	*h* = −8→6
Absorption correction: multi-scan (*CrysAlis PRO*; Agilent, 2011)	*k* = −8→5
*T*_min_ = 0.972, *T*_max_ = 0.986	*l* = −23→20
3236 measured reflections	

### Refinement

**Table:**

Refinement on *F*^2^	Primary atom site location: structure-invariant direct methods
Least-squares matrix: full	Secondary atom site location: difference Fourier map
*R*[*F*^2^ > 2σ(*F*^2^)] = 0.047	Hydrogen site location: inferred from neighbouring sites
*wR*(*F*^2^) = 0.123	H-atom parameters constrained
*S* = 1.05	*w* = 1/[σ^2^(*F*_o_^2^) + (0.0476*P*)^2^ + 0.27*P*] where *P* = (*F*_o_^2^ + 2*F*_c_^2^)/3
1956 reflections	(Δ/σ)_max_ = 0.001
136 parameters	Δρ_max_ = 0.30 e Å^−^^3^
0 restraints	Δρ_min_ = −0.34 e Å^−^^3^

### Fractional atomic coordinates and isotropic or equivalent isotropic displacement parameters (Å^2^)

**Table:**

	*x*	*y*	*z*	*U*_iso_*/*U*_eq_	Occ. (<1)
O1	0.9944 (2)	0.2500	0.34929 (8)	0.0164 (3)	
O2	1.7026 (2)	0.2500	0.67325 (10)	0.0406 (6)	
O3	1.4757 (2)	0.2500	0.74600 (9)	0.0243 (4)	
N1	0.6690 (3)	0.2500	0.33989 (10)	0.0178 (4)	
N2	0.7505 (3)	0.2500	0.41856 (10)	0.0171 (4)	
N3	1.5294 (3)	0.2500	0.68283 (11)	0.0216 (4)	
C1	0.8170 (3)	0.2500	0.30226 (12)	0.0156 (4)	
C2	0.8215 (3)	0.2500	0.21783 (12)	0.0148 (4)	
C3	0.9314 (2)	0.4411 (2)	0.19610 (9)	0.0195 (4)	
H3A	0.8624	0.5649	0.2101	0.023*	
H3B	1.0672	0.4426	0.2245	0.023*	
C4	0.9390 (2)	0.4400 (2)	0.10939 (9)	0.0204 (4)	
H4A	1.0100	0.5642	0.0953	0.024*	
C5	1.0483 (3)	0.2500	0.08863 (13)	0.0227 (5)	
H5A	1.0563	0.2500	0.0332	0.027*	
H5B	1.1844	0.2500	0.1168	0.027*	
C6	0.6100 (3)	0.2500	0.17372 (12)	0.0184 (5)	
H6A	0.5382	0.1279	0.1875	0.022*	0.50
H6B	0.5382	0.3721	0.1875	0.022*	0.50
C7	0.6192 (3)	0.2500	0.08724 (12)	0.0198 (5)	
H7	0.4821	0.2500	0.0586	0.024*	
C8	0.7279 (2)	0.0604 (3)	0.06587 (9)	0.0214 (4)	
H8A	0.7318	0.0591	0.0101	0.026*	
H8B	0.6572	−0.0632	0.0790	0.026*	
C10	0.9399 (3)	0.2500	0.42055 (12)	0.0148 (4)	
C11	1.0933 (3)	0.2500	0.48726 (12)	0.0148 (4)	
C12	1.2920 (3)	0.2500	0.47852 (12)	0.0184 (5)	
H12A	1.3293	0.2500	0.4288	0.022*	
C13	1.4351 (3)	0.2500	0.54269 (13)	0.0206 (5)	
H13A	1.5712	0.2500	0.5375	0.025*	
C14	1.3771 (3)	0.2500	0.61451 (12)	0.0166 (5)	
C15	1.1807 (3)	0.2500	0.62466 (12)	0.0163 (5)	
H15A	1.1445	0.2500	0.6745	0.020*	
C16	1.0384 (3)	0.2500	0.56058 (12)	0.0163 (5)	
H16A	0.9026	0.2500	0.5663	0.020*	

### Atomic displacement parameters (Å^2^)

**Table:**

	*U*^11^	*U*^22^	*U*^33^	*U*^12^	*U*^13^	*U*^23^
O1	0.0157 (8)	0.0229 (8)	0.0104 (7)	0.000	0.0019 (6)	0.000
O2	0.0151 (9)	0.0839 (17)	0.0224 (9)	0.000	0.0019 (7)	0.000
O3	0.0265 (9)	0.0349 (10)	0.0117 (8)	0.000	0.0031 (7)	0.000
N1	0.0194 (9)	0.0220 (10)	0.0123 (9)	0.000	0.0030 (7)	0.000
N2	0.0190 (10)	0.0210 (10)	0.0115 (9)	0.000	0.0024 (7)	0.000
N3	0.0197 (10)	0.0301 (11)	0.0149 (9)	0.000	0.0025 (8)	0.000
C1	0.0162 (10)	0.0159 (10)	0.0143 (10)	0.000	0.0011 (8)	0.000
C2	0.0153 (10)	0.0186 (11)	0.0107 (9)	0.000	0.0027 (8)	0.000
C3	0.0245 (8)	0.0199 (8)	0.0142 (7)	−0.0043 (7)	0.0036 (6)	−0.0010 (6)
C4	0.0259 (8)	0.0210 (8)	0.0147 (7)	−0.0066 (7)	0.0048 (6)	0.0019 (6)
C5	0.0178 (11)	0.0383 (14)	0.0128 (10)	0.000	0.0049 (9)	0.000
C6	0.0165 (11)	0.0256 (12)	0.0131 (10)	0.000	0.0024 (9)	0.000
C7	0.0180 (11)	0.0273 (12)	0.0138 (10)	0.000	0.0008 (9)	0.000
C8	0.0291 (9)	0.0222 (8)	0.0129 (7)	−0.0049 (7)	0.0030 (7)	−0.0030 (6)
C10	0.0188 (11)	0.0149 (10)	0.0116 (10)	0.000	0.0049 (8)	0.000
C11	0.0164 (11)	0.0152 (10)	0.0127 (10)	0.000	0.0023 (8)	0.000
C12	0.0191 (11)	0.0243 (12)	0.0126 (10)	0.000	0.0049 (9)	0.000
C13	0.0152 (11)	0.0296 (13)	0.0173 (11)	0.000	0.0033 (9)	0.000
C14	0.0174 (11)	0.0185 (11)	0.0133 (10)	0.000	0.0002 (8)	0.000
C15	0.0201 (11)	0.0172 (11)	0.0125 (10)	0.000	0.0051 (8)	0.000
C16	0.0162 (11)	0.0189 (11)	0.0145 (10)	0.000	0.0042 (8)	0.000

### Geometric parameters (Å, º)

**Table:**

O1—C10	1.365 (2)	C7—C8^i^	1.527 (2)
O1—C1	1.368 (3)	C7—C8	1.527 (2)
O2—N3	1.223 (2)	C7—H7	1.0000
O3—N3	1.226 (2)	C8—C4^i^	1.535 (2)
C1—N1	1.292 (3)	C8—H8A	0.9900
C1—C2	1.497 (3)	C8—H8B	0.9900
C2—C3^i^	1.5412 (19)	C10—N2	1.293 (3)
C2—C3	1.5412 (19)	C10—C11	1.460 (3)
C2—C6	1.542 (3)	C11—C12	1.392 (3)
C3—C4	1.541 (2)	C11—C16	1.402 (3)
C3—H3A	0.9900	C12—C13	1.386 (3)
C3—H3B	0.9900	C12—H12A	0.9500
C4—C5	1.528 (2)	C13—C14	1.385 (3)
C4—C8^i^	1.535 (2)	C13—H13A	0.9500
C4—H4A	1.0000	C14—C15	1.383 (3)
C5—C4^i^	1.528 (2)	C14—N3	1.475 (3)
C5—H5A	0.9900	C15—C16	1.382 (3)
C5—H5B	0.9900	C15—H15A	0.9500
C6—C7	1.539 (3)	C16—H16A	0.9500
C6—H6A	0.9900	N1—N2	1.421 (2)
C6—H6B	0.9900		
			
C10—O1—C1	102.83 (16)	C8—C7—C6	109.77 (12)
N1—C1—O1	112.43 (18)	C8^i^—C7—H7	109.3
N1—C1—C2	130.2 (2)	C8—C7—H7	109.3
O1—C1—C2	117.35 (17)	C6—C7—H7	109.3
C1—C2—C3^i^	109.31 (12)	C7—C8—C4^i^	109.56 (14)
C1—C2—C3	109.31 (12)	C7—C8—H8A	109.8
C3^i^—C2—C3	109.10 (17)	C4^i^—C8—H8A	109.8
C1—C2—C6	110.42 (17)	C7—C8—H8B	109.8
C3^i^—C2—C6	109.34 (12)	C4^i^—C8—H8B	109.8
C3—C2—C6	109.34 (12)	H8A—C8—H8B	108.2
C4—C3—C2	109.45 (13)	N2—C10—O1	112.58 (18)
C4—C3—H3A	109.8	N2—C10—C11	128.51 (19)
C2—C3—H3A	109.8	O1—C10—C11	118.91 (18)
C4—C3—H3B	109.8	C12—C11—C16	120.1 (2)
C2—C3—H3B	109.8	C12—C11—C10	120.67 (18)
H3A—C3—H3B	108.2	C16—C11—C10	119.19 (19)
C5—C4—C8^i^	109.71 (14)	C13—C12—C11	119.7 (2)
C5—C4—C3	109.36 (14)	C13—C12—H12A	120.2
C8^i^—C4—C3	109.38 (13)	C11—C12—H12A	120.2
C5—C4—H4A	109.5	C12—C13—C14	119.1 (2)
C8^i^—C4—H4A	109.5	C12—C13—H13A	120.4
C3—C4—H4A	109.5	C14—C13—H13A	120.4
C4^i^—C5—C4	109.59 (17)	C15—C14—C13	122.3 (2)
C4^i^—C5—H5A	109.8	C15—C14—N3	118.57 (19)
C4—C5—H5A	109.8	C13—C14—N3	119.13 (19)
C4^i^—C5—H5B	109.8	C16—C15—C14	118.46 (19)
C4—C5—H5B	109.8	C16—C15—H15A	120.8
H5A—C5—H5B	108.2	C14—C15—H15A	120.8
C7—C6—C2	109.26 (17)	C15—C16—C11	120.3 (2)
C7—C6—H6A	109.8	C15—C16—H16A	119.8
C2—C6—H6A	109.8	C11—C16—H16A	119.8
C7—C6—H6B	109.8	C1—N1—N2	106.17 (18)
C2—C6—H6B	109.8	C10—N2—N1	105.99 (17)
H6A—C6—H6B	108.3	O2—N3—O3	123.5 (2)
C8^i^—C7—C8	109.38 (18)	O2—N3—C14	118.08 (18)
C8^i^—C7—C6	109.77 (12)	O3—N3—C14	118.39 (18)
			
C10—O1—C1—N1	0	N2—C10—C11—C12	180
C10—O1—C1—C2	180	O1—C10—C11—C12	0
N1—C1—C2—C3^i^	−120.32 (12)	N2—C10—C11—C16	0
O1—C1—C2—C3^i^	59.68 (12)	O1—C10—C11—C16	180
N1—C1—C2—C3	120.32 (12)	C16—C11—C12—C13	0
O1—C1—C2—C3	−59.68 (12)	C10—C11—C12—C13	180
N1—C1—C2—C6	0	C11—C12—C13—C14	0
O1—C1—C2—C6	180	C12—C13—C14—C15	0
C1—C2—C3—C4	179.20 (14)	C12—C13—C14—N3	180
C3^i^—C2—C3—C4	59.7 (2)	C13—C14—C15—C16	0
C6—C2—C3—C4	−59.83 (17)	N3—C14—C15—C16	180
C2—C3—C4—C5	−60.23 (18)	C14—C15—C16—C11	0
C2—C3—C4—C8^i^	59.94 (17)	C12—C11—C16—C15	0
C8^i^—C4—C5—C4^i^	−59.3 (2)	C10—C11—C16—C15	180
C3—C4—C5—C4^i^	60.7 (2)	O1—C1—N1—N2	0
C1—C2—C6—C7	180	C2—C1—N1—N2	180
C3^i^—C2—C6—C7	−59.70 (11)	O1—C10—N2—N1	0
C3—C2—C6—C7	59.70 (11)	C11—C10—N2—N1	180
C2—C6—C7—C8^i^	−60.13 (12)	C1—N1—N2—C10	0
C2—C6—C7—C8	60.13 (12)	C15—C14—N3—O2	180
C8^i^—C7—C8—C4^i^	60.1 (2)	C13—C14—N3—O2	0
C6—C7—C8—C4^i^	−60.40 (18)	C15—C14—N3—O3	0
C1—O1—C10—N2	0	C13—C14—N3—O3	180
C1—O1—C10—C11	180		

Symmetry code: (i) *x*, −*y*+1/2, *z*.

### Hydrogen-bond geometry (Å, º)

**Table:**

*D*—H···*A*	*D*—H	H···*A*	*D*···*A*	*D*—H···*A*
C13—H13*A*···N2^ii^	0.95	2.59	3.297 (3)	132
C16—H16*A*···O2^iii^	0.95	2.49	3.256 (3)	137

Symmetry codes: (ii) *x*+1, *y*, *z*; (iii) *x*−1, *y*, *z*.
